# Biomedical Applications of Chitosan and Its Derivative Nanoparticles

**DOI:** 10.3390/polym10040462

**Published:** 2018-04-23

**Authors:** Dongying Zhao, Shuang Yu, Beini Sun, Shuang Gao, Sihan Guo, Kai Zhao

**Affiliations:** Key Laboratory of Microbiology, School of Life Science, Heilongjiang University, Harbin 150080, China; zhaody823@126.com (D.Z.); yushuang1199@163.com (S.Y.); sbn1031@126.com (B.S.); 15545956067@163.com (S.G.); guosihan926@126.com (S.G.)

**Keywords:** chitosan and its derivatives, nanoparticles, delivery system, biomedical application

## Abstract

Chitosan is a biodegradable natural polymer with many advantages such as nontoxicity, biocompatibility, and biodegradability. It can be applied in many fields, especially in medicine. As a delivery carrier, it has great potential and cannot be compared with other polymers. Chitosan is extremely difficult to solubilize in water, but it can be solubilized in acidic solution. Its insolubility in water is a major limitation for its use in medical applications. Chitosan derivatives can be obtained by chemical modification using such techniques as acylation, alkylation, sulfation, hydroxylation, quaternization, esterification, graft copolymerization, and etherification. Modified chitosan has chemical properties superior to unmodified chitosan. For example, nanoparticles produced from chitosan derivatives can be used to deliver drugs due to their stability and biocompatibility. This review mainly focuses on the properties of chitosan, chitosan derivatives, and the origin of chitosan-based nanoparticles. In addition, applications of chitosan-based nanoparticles in drug delivery, vaccine delivery, antimicrobial applications, and callus and tissue regeneration are also presented. In summary, nanoparticles based on chitosan have great potential for research and development of new nano vaccines and nano drugs in the future.

## 1. Introduction

Polymer nanoparticles are extensively applied in the biomedical field as tools in the diagnosis and treatment of diseases [1]. As a delivery carrier, polymer nanoparticles can adsorb to or be loaded with multiple drugs and can more effectively control the release of drugs. Additionally, polymer nanoparticles can encapsulate drugs on their surfaces. The ability of these polymer-based nanoparticles to target molecules with specific receptors on the cell surface and also to enter cells can aid in a more secured and efficient delivery of targeted drugs and in gene therapy [2]. Polymer nanoparticles, especially those with hydrophilic surfaces, are widely used as carriers due to their very small nonspecific protein adsorption properties. Also, they can be used for the diagnosis and treatment of complicated diseases. Chitosan is a naturally occurring polymer that is abundant in nature. Due to its good physicochemical properties and unique biological properties, chitosan finds applications in many industries, including the medical, food, chemical, cosmetics, water treatment, metal extraction and recovery, biochemical, and biomedical engineering industries. However, chitosan is not soluble in aqueous solutions, a major disadvantage that limits its widespread application in living systems [3]. However, chitosan has some functional groups that allow for graft modification that imparts the modified chitosan with special properties. Such modifications can be employed to chemically modify chitosan to improve its solubility and consequently widen its applications. These chemical modifications produce many kinds of chitosan derivatives that have sustained-release properties and are nontoxic, biocompatible, and biodegradable [4]. Furthermore, chitosan nanoparticles can improve the body’s immune function to achieve antitumor activity [5]. Due to their good biocompatibility and biodegradability and their ease of modification, chitosan nanoparticles are used as drug carriers [6]. Chitosan nanoparticles have an extensive use in drug and vaccine delivery, as vaccine adjuvant, as an antimicrobial, in tissue engineering, and in other applications.

## 2. Chitosan Properties

Chitosan is a linear homopolymer that is constituted of β-(1,4)-linked *N*-acetyl-glucosamine units [7,8,9]. It is a partially deacetylated polymer acquired from basic deacetylation of chitin, which is a glucose-based unbranched polysaccharide that is found extensively in the major components of crustaceans and insect exoskeletons, as well as some bacterial and fungal cell walls [10]. The quality of chitosan depends on the source of chitin and its separation and the degree of deacetylation of chitin [11]. Chitosan has excellent biological properties, including being nontoxic, mucoadhesive, hemocompatible, biodegradable, and possessing antitumor, antioxidant, and antimicrobial properties. These properties make chitosan a very attractive biomaterial for different applications in the biomedical field.

### 2.1. Nontoxicity

One of the main characteristics of chitosan is that it does not induce intense inflammation or provoke the body’s immune response system. Research has shown chitosan with different molecular weights and degrees of deacetylation to exhibit a low level of toxicity that is similar to that of succinyl-derived chitosan and chitosan nanoparticles [12,13,14,15].

### 2.2. Antimicrobial Activities

Usually, due to the catatonic nature of the polymer, chitosan solutions have bactericidal and bacteriological properties. Positive charge on the polymer chain will adhere to bacterial surfaces, inducing changes in the permeability of the membrane wall that prevents microbial growth [16].

Low degree of deacetylation and low pH chitosan has better antibacterial activity. Reducing the molecular weight can increase the antibacterial activities against gram-negative bacteria and decrease the activities against gram-positive bacteria. In addition, chitosan has a broad extent of antimicrobial activities against gram-positive and gram-negative bacteria, with a high killing rate through the interaction between chitosan and its derivatives and the bacterial cell wall [17]. This interaction between chitosan and the bacterial cell is dependent on hydrophilicity of the cell wall, which may explain chitosan’s lower toxicity to mammalian cells [18].

### 2.3. Mucoadhesivity

Chitosan’s ability to adhere to surfaces is one of its major features. This feature does not only generate new approaches to deliver beneficial molecules through mucosal pathways, but also helps to adsorb molecules that have no affinity for mucus [19]. By means of permeation, chitosan enhances adhesivity of polymers, which is helpful to open the tight epithelial junction [20].

### 2.4. Hemocompatibility

Chitosan has been widely used in studies related to coagulation. In fact, chitosan can speed the rate of wound healing through interactions between platelet and amino groups on chitosan [21]. The hemostatic properties of chitosan have been widely used in wound healing. As a material for wound dressing, chitosan has several features, such as chemoattraction, activation of macrophages and neutrophils, acceleration of granulation tissue and re-epithelization, limited scar formation and contraction, analgesic properties, hemostasis, and intrinsic antibacterial properties [22].

### 2.5. Antitumor Activity

Recent surveys have shown that chitosan and its derivatives have antitumor activities using both in vitro and in vivo models. The antitumor effect of chitosan derivatives is caused by an increase in the secretion of interleukin (IL)-1 and 2, which results in the maturation and infiltration of cytolytic T-lymphocytes [23].

### 2.6. Antioxidant Activity

It is well-known that antioxidants have beneficial effects on health. They prevent destruction of membrane lipids, proteins, and DNA by the body’s reactive oxygen radical molecules [24]. Studies have shown that chitosan and its derivatives have the ability to scavenge the active oxygen free radicals in vitro. Low-weight chitosan molecules have several advantages over high-weight chitosan molecules in the elimination of free radicals [25]. One study suggested that the mechanism of chitosan’s antioxidant activity may be through the stabilization of the free radicals by amino and carboxyl groups on chitosan [26].

### 2.7. Biodegradability

Chitosan in biological organisms can be catalyzed by bioenzymes to depolymerize the molecule. The degradation products are *N*-acetyl glucose and glucosamine, which are nontoxic to the human body. Degradation intermediates do not accumulate in the body and have no immunogenicity.

## 3. Chitosan Derivatives

Chitosan has active hydroxyl and amino groups that can undergo various chemical reactions including hydroxylation, carboxylation, alkylation, acylation, and esterification. These reactions introduce pendant groups into the chitosan, destroying the crystal structure of chitosan and consequently increasing the solubility of the modified chitosan. These chitosan derivatives with improved physicochemical and biological properties are better suited for use as carriers in the biomedical field [27].

### 3.1. Alkylated Chitosan

**Figure 1:**
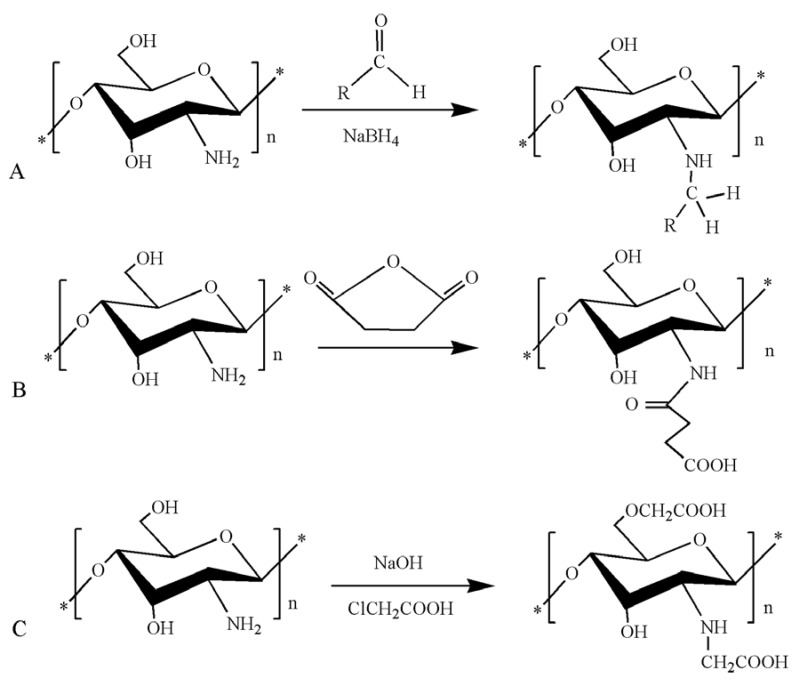
Synthetic route of chitosan derivatives. (**A**) *N*-alkylated chitosan; (**B**) *N*-succinylated chitosan; (**C**) carboxymethyl chitosan.

Both the functional groups –NH_2_ (amino) and C_3_, C_6_–OH (hydroxyl) can be involved in chitosan alkylation. However, reactions involving the amino group occur at higher rate in comparison to those involving the hydroxyl groups and also better protect special functional groups. Hence, chitosan alkylation occurs mainly through the amino group to generate *N*-alkylated chitosan derivatives [28]. A series of *N*-alkylated chitosan molecules were synthesized by Chen. The reaction scheme is as presented in Figure 1A. Hemolysis and toxicity assays showed that *N*-alkylated chitosan has good biocompatibility. From results of in vitro blood coagulation tests, *N*-alkylated chitosan had better hemostatic activity than unmodified chitosan [29]. Hydrogen bonding between chitosan molecules is significantly reduced by the presence of the alkyl groups, making the modified alkylated chitosan more water soluble and more promising in biomedical applications.

### 3.2. Acylated Chitosan

The –NH_2_ and –OH groups on the chitosan molecule can participate in an ester or amide reaction with organic acid anhydride or organic acid chloride. When preparing acylated chitosan, attention needs to be paid to the reaction temperature and the type of catalyst employed. The solubility of acylated chitosan in water or in an organic solvent is generally improved by introducing different molecular weights of fats or aromatic acyl groups. In one study, *N*-succinylated chitosan was generated through the introduction of succinyl in the *N*-position of the chitosan glucosamine unit [30]. The reaction scheme is as presented in Figure 1B. *N*-succinylated chitosan molecules contain COOH, C_2_–NH_2_; C_3_–OH, C_6_–OH, and other active groups, which allow it to better adsorb divalent copper ions. Acylated chitosan has good processing properties and a sustained-release effect. It is a new type of auxiliary material that can be used for oral insoluble skeletal formulations.

### 3.3. Carboxylated Chitosan

In order to obtain carboxyl-modified chitosan derivatives, carboxylated chitosan reactions generally occur through both the –NH_2_ and –OH. Carboxylation can be achieved using glyoxylic acid. Chitosan has been treated with monochloroacetic acid under different conditions to obtain carboxymethyl chitosan. The reaction scheme is as presented in Figure 1C. The water solubility of carboxymethyl chitosan is dependent upon the conditions of modification and the degree of carboxymethylation [31]. The carboxylation of chitosan not only improves the water solubility of chitosan, but also generates amphiphilic chitosan derivatives with both –NH_2_ and –COOH groups. These derivatives have good water solubility and surface activity, as well as film-forming, moisture absorption, moisture retention, antibacterial [32], antioxidant [33], and other biological properties which render them useful for various applications in cosmetics, food, and medical industry.

### 3.4. Quaternary Ammonium Chitosan

Chitosan quaternization reactions can occur through both the –NH_2_ and –OH groups. Quaternization generally involves reaction of chitosan with methyl iodide, although it may involve chemicals other than methyl iodide [34,35,36]. The derivatives are synthesized by chitosan and quaternary epoxides; it is possible to prepare cationized derivatives (quaternary ammonium chitosan) with diverse hydrophobicity/hydrophilicity through the various alkyl chains on quaternary epoxides [37].

**Figure 2:**
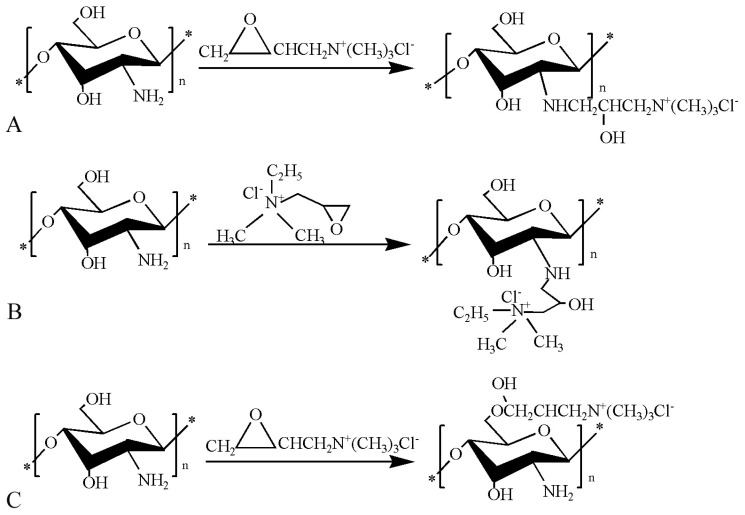
Synthetic route of chitosan derivatives. (**A**) *N*-2-Hydroxypropyl trimethyl ammonium chloride chitosan; (**B**) *N*-2-hydroxypropyl dimethyl ethyl ammonium chloride chitosan; (**C**) *O*-2′-hydroxypropyltrimethyl ammonium chloride chitosan.

Our group synthesized *N*-2-hydroxypropyl trimethyl ammonium chloride chitosan (*N*-2-HACC), *N*-2-hydroxypropyldimethyl ethyl ammonium chloride chitosan (*N*-2-HFCC), and *O*-2′-hydroxypropyltrimethyl ammonium chloride chitosan (*O*-2′-HACC). The reaction schemes are as presented in Figure 2A–C. *N*-2-HACC had greater stability and solubility, better antibacterial activities, and lower toxicity compared to chitosan [38]. Compared with *N*-2-HACC nanoparticles, *N*-2-HFCC nanoparticles exhibited higher loading ability and embedding ratio when used to encapsulate vaccine antigens. *N*-2-HFCC can adhere on the mucosal surface of the respiratory tract, gastrointestinal tract, and urinary tract, which promotes the absorption of *N*-2-HFCC and induces mucosal immunoreactivity: this expands the range chitosan application [39]. The oxygen on chitosan C6 was replaced by 2,3-epoxypropyltrimethyl ammonium chloride to form *O*-2′-HACC. *O*-2′-HACC had excellent water solubility, which is attributed to the presence of hydrophilic groups. In addition, *O*-2′-HACC had high antibacterial activity, good security, and was nontoxic [40]. Quaternized chitosan has considerable application in the preparation of anticoagulant materials, functional protein materials, and functional polymers due to its high water solubility and safety [41].

### 3.5. Esterified Chitosan

**Figure 3:**
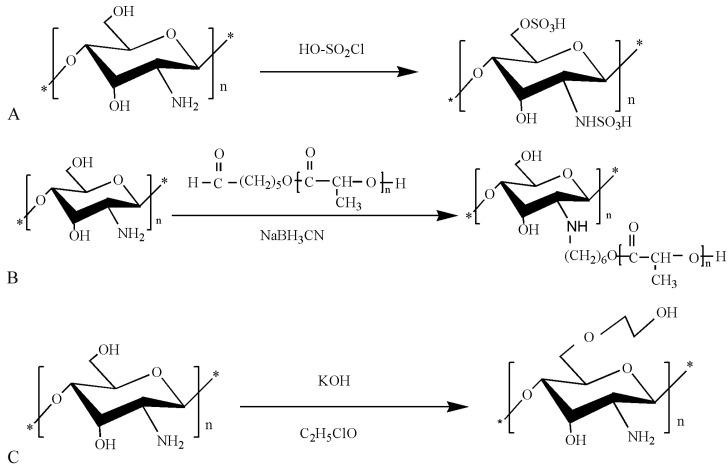
Synthetic route of chitosan derivatives. (**A**) Sulfated chitosan; (**B**) chitosan-*g*-oligo (L-lactic acid); (**C**) hydroxyethyl chitosan.

The esterification of chitosan occurs with some of the oxygen-containing inorganic acids (or their anhydrides) on the chitosan molecule. Sulfated chitosan has a wide range of applications as substitutes for heparin or heparin sulfate in the field of biology, including as anticoagulant and as antiviral drugs, to promote osteogenic differentiation and specific binding of proteins [42,43,44]. The regulatory mechanism of sulfated chitosan is the same as heparin. In vivo studies show that the activity of proteins and cells is influenced by violent reaction with specialized cells and biologically active compounds [45,46]. Sulfated chitosan was successfully prepared, and the reaction scheme is as presented in Figure 3A [47]. Chitosan derivatives obtained through esterification can be used for high-strength fibers.

### 3.6. Graft Copolymer Chitosan

Chitosan graft copolymerization imparts some new excellent properties to chitosan through the introduction of other side chain groups. The resulting modified chitosan can be used to modify the surface of fabrics or cellulose and also improve the antibacterial properties of chitosan [48,49,50]. Modified chitosan obtained through graft modification can also be used on the surface of tissue-engineering materials to improve the anticoagulant properties [51,52].

Chitosan can be coupled to oligo-lactic acid containing terminal aldehyde group to generate a graft copolymer that is soluble in *N,N*-dimethylformamide (DMSO), dimethyl sulfoxide dimethyl sulfoxide (DMF), and acetic acid. The reaction scheme is as presented in Figure 3B. The graft copolymerization of chitosan copolymer holds great promise for widespread use in the production of sustained-release drugs and other biopharmaceuticals [53].

### 3.7. Etherified Chitosan

Chitosan etherification reaction occurs through the –OH group on chitosan, leading to the formation of the corresponding alkylating agent (alkyne derivatives). The produced alkyne derivatives then undergo a deacetylation reaction to obtain chitosan ether derivatives. The reaction scheme is as presented in Figure 3C. Chitosan ether derivatives are not cytotoxic, do not have a marked influence on the growth of fibroblasts, and do not cause significant irritation, but they do cause delayed hypersensitivity and delayed inflammatory response [54]. Hydroxyethyl chitosan has excellent performance biocompatibility and biodegradability and is appropriate for applications in the medical field. They also have excellent bacteriostatic and hygroscopic moisturizing effects and are safe for use as natural textile softening and finishing agent. Hydroxyethyl chitosan can also be used as a preservative in cosmetics where they exhibit antibacterial effects on common bacteria such as *Escherichia coli.*

## 4. Chitosan-Based Nanoparticles

Chitosan-based nanoparticles possess large numbers of lone-pair electrons and have high binding power with material with empty orbital. They are used in drugs and gene delivery [55,56], in biosensors [57], and in fractionated images [58,59]. This function of chitosan-based nanoparticles is based on the uniformity and particle size of the prepared microspheres. Particle size affects the amount of antigen adsorption and distribution, which affects the immune effect. The structure of the microspheres, the size of surface micropores, and the release rate of antigen affect the function of microspheres. Chitosan nanoparticles were obtained through emulsion crosslinking, ionically crosslinking, solvent evaporation, spray drying, precipitation, or flocculation and chitosan solution coating.

### 4.1. Emulsion Crosslinking

**Figure 4:**
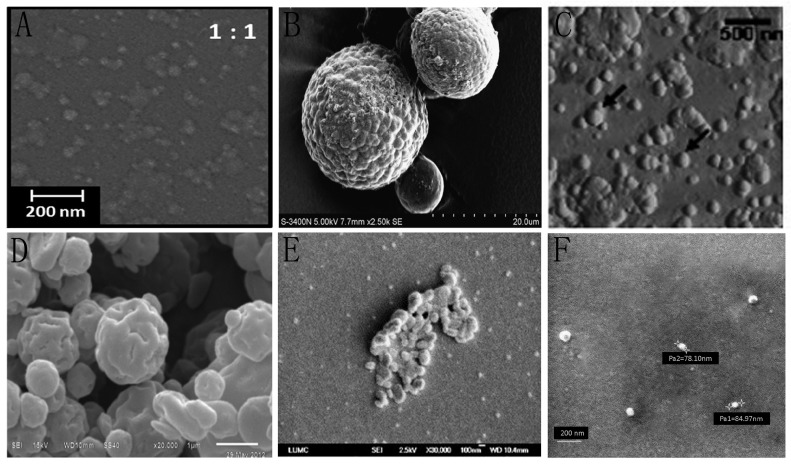
Scanning electron microscopy (SEM) or scanning force microscopy (SFM) micrographs of synthesized chitosan nanoparticles using six different techniques. (**A**) SEM micrograph of the nanoparticles modified through emulsion crosslinking. Reproduced with permission from [61]; (**B**) SEM micrograph of the nerve growth factor-loaded chitosan nanoparticles modified through ionically crosslinking. Reproduced with permission from [62]; (**C**) SFM micrograph of the chitosan-modified (poly(d,l-lactide-*co*-glycolide); PLGA) nanoparticles modified through solvent evaporation. Reproduced with permission from [63]; (**D**) SEM micrograph of the cellulose-chitosan complex nanoparticles modified through spray drying. Reproduced with permission from [64]; (**E**) SEM micrograph of the nanoparticles modified through precipitation, reproduced with permission from [66]; (**F**) SEM micrograph of the chitosan-alginate nanoparticles modified through chitosan solution coating. Reproduced with permission from [67].

The nanoparticles prepared by the emulsion crosslinking method use chitosan as the polymer and tripolyphosphate as the crosslinking agent. In this way, chitosan nanoparticles are produced by the reaction between the negative groups of sodium tripolyphosphate and the positively charged amino (–NH_2_) groups on chitosan [60]. Regarding the morphology of the nanoparticles modified through emulsion crosslinking, scanning electron microscopy (SEM) photomicrographs are as presented in Figure 4A [61]. Nanoparticles shown are regular spherical-shaped, narrowly distributed particles. In addition, these nanoparticles have improved properties, such as better stability and prolonged drug release time.

### 4.2. Ionically Crosslinked

Ionically crosslinking to produce nanoparticles involves a reaction between chitosan and sodium tripolyphosphate or sodium metaphosphate as the ionic crosslinking agent. The nanoparticles are produced by ionic interaction between amino groups of chitosan and phosphoric groups of tripolyphosphate. Regarding the morphology of the nerve growth factor-loaded chitosan nanoparticles modified through ionically crosslinking, SEM photomicrographs are as presented in Figure 4B [62]. The nanoparticles have a rough surface, suitable particle size distribution, high trapping efficiency, and good a drug-loading rate.

### 4.3. Solvent Evaporation

The solvent evaporation technique of preparing nanoparticles is based on the difference in volatility of the solute in the dissolving phase combined with sonication. The obtained chitosan derivatives tend to have amphipathic properties. After mixing with the oil phase and anticancer drug and distilling off the organic solvent by sonication, the carrier-loaded chitosan derivative nanoparticle is obtained. Regarding the morphology of the chitosan-modified poly (d, l-lactide-*co*-glycolide) (PLGA) nanoparticles modified through solvent evaporation, scanning force microscopy (SFM) photomicrographs are as presented in Figure 4C [63]. The nanoparticles have a smooth surface and a spherical well-defined shape.

### 4.4. Spray Drying

The spray drying method of preparing chitosan nanoparticles involves first dissolving the drug and chitosan together in a solvent. The resulting solution is sprayed through a nozzle into a drying chamber to form small droplets, which contains hot air to evaporate water and volatile organic solvents in the droplets to obtain the nanoparticles. When utilized as a carrier for neurotrophic factors, nanoparticles are produced from a complex of ethyl cellulose and chitosan by the spray drying method. Regarding the morphology of the nanoparticles, SEM photomicrographs are as presented in Figure 4D [64]. The nanoparticles have a uniform and spherical shape. These nanoparticles were found to have sustained release; such nanoparticles could play a significant role in the treatment of neurodegenerative disorders and pulmonary tuberculosis.

### 4.5. Precipitation or Flocculation

Nanoparticles can be prepared by precipitation or flocculation using sodium sulfate as the precipitating agent. The extent of precipitation is dependent on the concentration of sodium sulfate [65]. Regarding the morphology of the nanoparticles modified through precipitation, SEM photomicrographs are as presented in Figure 4E [66]. The nanoparticles have nonuniform particle sizes, and one possible explanation for these findings is that they were subjected to the freeze-dry process for sample preparation for the SEM. 

### 4.6. Chitosan Solution Coating

Chitosan solution coating is produced by adding the existing nanoparticles to a suitable concentration of chitosan solution. The nanoparticles become covered with a moderate shell of chitosan due to chitosan’s adhesiveness and the presence of lone-pair electrons. Regarding the morphology of the chitosan-alginate nanoparticles modified through chitosan solution coating, SEM photomicrographs are as presented in Figure 4F [67]. The nanoparticles have a smooth surface and good shape, with particle sizes ranging between 75 and 85 nm. In addition, these nanoparticles have good absorption and good target-controlled release performance.

## 5. Applications of Chitosan-Based Nanoparticles in Drug Delivery

### 5.1. Antitumor Drug Delivery

Doxorubicin is commonly used for cancer treatment but produces unwanted side effects such as cardiotoxicity. In order to reduce these side effects, the drug has been encapsulated in chitosan nanoparticles. These nanoparticles can improve the absorption of doxorubicin in the whole small intestine [68]. The nanoparticle increases survival time of drug conjugates or the free drug and also reduces adverse reactions of drugs [69]. Chitosan tripolyphosphate (TPP) nanoparticles can adhere to and help retain drugs such as doxorubicin on mucosal surfaces. One study has shown 46% chitosan/TPP nanoparticles to be preserved in rat colon after incubation for 2 h at 37 °C, causing mucoadhesive activity of doxorubicin to be increased from 1.88% to 38.74% [70].

In a recent study involving 5-fluorouracil-based chitosan nanoparticles, the nanoparticles reduced the diffusion of HT29 (human colorectal adenocarcinoma) and PC-3 (human prostate-3) tumor cells, and also restrained their adhesivity to human umbilical vein endothelial cells [71].

Lung cancer is one of the most frequent causes of cancer death in developed countries [72]. Almost 80% of all these lung cancers are non-small cell lung cancers. Treatment of lung cancer with paclitaxel (a chemotherapy drug) reveals apparent activity for non-small cell lung cancer at later stages. The transient stimulation of blood flow by intracellular nanoparticle aggregates, leading to enhanced trapping ability in pulmonary capillaries, has been established as the mechanism by which nanoparticles containing paclitaxel destroys lung tumor [73]. In addition, the authors also showed that, under acidic tumor conditions, nanoparticles containing paclitaxel become more aggressive and strongly interact with negatively charged tumor cells [73].

### 5.2. Protein and Peptide Drug Delivery

Protein-based drugs are easily hydrolyzed by enzymes in the gastrointestinal tract. However, when these drugs are encased in chitosan nanoparticles, they are not easily damaged by gastric enzymes. In addition, chitosan nanoparticles can significantly enhance the stability of the drug. Chitosan nanoparticles control drug release, improve the biodegradation of proteins, and enhance the assimilation of hydrophilic substances through the epithelial layer. They are being researched for the delivery of drugs that exert their action in the stomach [74].

Insulin-based chitosan nanoparticles have been synthesized through membrane emulsification and crosslinking. The resulting chitosan nanoparticles exhibited high drug entrapment efficiency, good stabilization, low outbreak, and steady release of insulin [75].

Chitosan was successfully crosslinked with poly (ethylene glycol) dialdehyde, forming a hydrogel that enhances protein release. Therefore, synthetic poly (ethylene glycol) chitosan derivatives may be suitable as carriers for the controlled release of proteins and other large biological molecules that are used in oral drugs [76].

### 5.3. Gene Delivery

Chitosan can bind DNA and prevent DNA from being degraded by nucleases, thereby increasing the resident time of DNA in the gastrointestinal tract [77,78,79]. Chitosan has potential adjuvant properties, such as the promotion of endocytosis and increased immune response [80].

Plasmid DNA encapsulated in chitosan nanoparticles was produced by an intricate coagulation process, and the results showed that the plasmid DNA was effectively enclosed in chitosan nanoparticles and expressed in vivo [81]. This may be a beneficial way to improve expression and control of interleukine-2 (IL-2)-encoding genes encapsulated in chitosan nanoparticles. Chitosan nanoparticles loaded with IL-2 expression plasmids have been evaluated for gene-based immune therapy. The results showed that the plasmid remained unchanged during encapsulation. High levels of chitosan nanoparticles loaded with IL-2 expression plasmids were obtained and showed similar production of IL-2 liposomes. The molecular weight and mass quantity of chitosan affects IL-2-producing cells in vitro [82]. Two different DNA plasmids (pGL2 and pMK3) encapsulated in chitosan nanoparticles remained unchanged both in their structure and function [83].

### 5.4. Antibiotic Delivery

Chitosan-encapsulated gentamicin with both antimicrobial and antioxidant activities has been prepared for lung delivery [84]. Since fucoidan (sulfated polysaccharide in seaweed) has antioxidants to remove the active oxygen generated by gentamicin [85], a study has been performed to examine the release properties of nanoparticles produced by encapsulating gentamicin and fucoidan in chitosan. The produced nanoparticles were found to have increased antimicrobial activity and also reduced systemic toxicity, indicating promise for the treatment of *Pneumonia* infections.

### 5.5. Polyphenol Delivery

Although dietary polyphenols show diverse pharmacological potential—such as antioxidant properties; anti-inflammatory effects; and prevention of cardiovascular disease, cancer, and Alzheimer’s disease—their slow rates of assimilation and poor bioavailability prevent the use of these chemical substances as orally administered therapeutic agents [86]. In order to solve this problem, Liang et al. [87] encapsulated tea-derived polyphenols into chitosan nanoparticles for oral delivery. They concluded that chitosan nanoparticles can enhance the stability of tea polyphenols and guard against oxidation or deterioration in the gastrointestinal tract. Encapsulation also led to direct uptake of polyphenolic compounds at the tight epithelial junctions by epithelial cells through endocytosis.

Chitosan nanoparticles loaded with rosmarinic acid have been produced through an ionic gelation method for ocular administration. The nanoparticles did not show cytotoxicity to retinal pigment epithelium (ARPE-19) and human corneal (HCE-T) cell lines. It was found that the penetrability is improved by the enclosed rosmarinic acid in nanoparticles compared to the free solution. Study of mucoadhesion revealed that mucosal nanoparticles interacted with the eyes [88].

## 6. Applications of Chitosan-Based Nanoparticles in Vaccine Delivery

As a result of the mucoadhesive and osmotic properties of chitosan, chitosan can greatly enhance the adsorption and transport of peptides across the nasal epithelium [89]. Numerous researches have showed that chitosan can promote transport of macromolecules across the mucosal barrier and interacts with nasal tissue [84]. Chitosan microspheres can greatly improve the systemic and local immune response to diphtheria toxoid after nasal administration in mice [90].

Recently, oral delivery of glucomannan-modified chitosan nanoparticles was studied through in vitro and in vivo testing in mice. In this study, the lyophilized nanoparticles maintained the biological activity of mediators and blocked antigens. Glucuronidation of chitosan nanoparticles significantly induced systemic (serum immunoglobulin G (IgG) titers), mucosal (secretory immunoglobulin A (IgA)), and cell-mediated (IL-2 and interferon-γ (IFN-γ)) immune responses compared to unmodified chitosan nanoparticles [91].

The antibody (IgA)-based chitosan-dextran sulfate nanoparticles with pertussis toxin and assessing IgA were studied the results have shown that the prior absorption of IgA-based chitosan–dextran sulfate nanoparticles occurs through nasal membranes or M cells in mice following intranasal immunization in vivo [92].

*N*-2-HACC and *N*,*O*-carboxymethyl chitosan (CMC) nanoparticles have been synthesized and evaluated as vaccine adjuvant for Newcastle disease vaccine (NDV) and infectious bronchitis vaccine (IBV). The immune responses in chicken revealed that those nanoparticles containing NDV/IBV can induce better intranasal inoculation of IgG and IgA antibodies, and also enhance the proliferation of lymphocytes [93].

Chitosan is a biodegradable biopolymer that has the capacity to stimulate an immune response. In addition, chitosan nanoparticles in combination with plasmid DNA enhance antigen-specific immunity [94]. Some studies have investigated intranasal DNA vaccination. In addition, IFN-γ-generating T-cells were found in the lungs, and CD8+ and CD4+ T-cells can induce effectively specific immunological responses for the formulation of a DNA carrier with polyethyleneimine, upon which the metastasis rate of genes in the respiratory tract was improved 1000-fold [95].

## 7. Antimicrobial Activities

The antibacterial activities of chitosan and its derivatives are affected by the molecular weight, degree of deacetylation, pH of the solution, and the role of cells. The antimicrobial and other properties of chitosan (such as nontoxicity, biocompatibility, and biodegradability) make chitosan and its micro- and nanoparticles potentially useful in various fields.

Many warm-blooded animals are susceptible to parasites such as ticks and mites, and chitosan is the most effective insecticide for these parasites and certain bacteria and fungi. In order to prevent flies, mosquitoes, and other health pests, one of the commonly used means of prevention and control is spraying insecticides on doors and windows. However, wind or rain can gradually deplete the insecticide, reducing its efficacy and action time. The chitosan macromolecule can form a permeable water-insoluble film (but semipermeable membrane on the surface of the drug), which can prolong the persistence of insecticides and doubles the killing rate of flies.

To assess the feasibility of chitosan hydrogels to prevent breast cancer from infection during lactation, antibiotics are usually used to prohibit microbial infections. However, not only are their actions short term, but they could also promote antibiotic resistance. Therefore, researchers have measured the influence of injecting chitosan hydrogel into the teat. The process did not only prevent pathogens from entering the milk, but also accelerated the degradation of the pathogen in the breast. The injected chitosan hydrogel increases the degradation of galactophore and activates the immune response, which restrains the active microbial infection [96].

The antibacterial properties of chitosan are based on interaction between phosphoryl groups on chitosan and lipopolysaccharide on bacterial cell membrane. This antibacterial action of chitosan has the added benefit of preventing lung bacterial infections. Chitosan nanoparticles loaded with rifampin could lead to the stable release of the encapsulated drugs over a period of 24 h without toxicity to organs and cells. In vivo studies showed that the nanoparticles can improve the concentration of plasma to a maximum and prolong the mean retention time [97].

## 8. Callus and Tissue Regeneration

Chitosan and its derivatives exhibit biodegradability, biocompatibility, antibacterial activity, and low immunogenicity, which can accelerate the development of biological materials for wound healing [98]. They provide a three-dimensional tissue growth matrix, activate macrophage activity, and stimulate cell proliferation [99]. Chitosan facilitates activity of pronuclear leukocytes and activates macrophage fibroblasts to enhance granulation and to repair tissue [100]. Slow degradation of *N*-acetyl-β-d-glucosamine stimulates fibroblast proliferation, which results not only in the precipitation of collagen, but also the synthesis of hyaluronic acid in the wound. This accelerates wound healing and prevents scarring [101].

Sankar et al. [102] made a lyophilized glutaraldehyde crosslinked chitosan sponge for blood hemostasis. The chitosan acted as a mechanical barrier to blood, causing it to coagulate immediately*.*

Another type of composite particle, tricalcium phosphate-chitosan, has been used as a bone substitute and a tissue-engineering scaffold in order to obtain high bone formation efficacy. The nanoparticles had capabilities to fill some types defect sites packaging, act as potential bone substitute, improve drug release capacity, and serve as osteoblast cell culture scaffold [103].

## 9. Future Perspectives

Chitosan and its derived nanoparticles can be used as carrier materials for nano delivery systems and have many biomedical applications, such as drug delivery, vaccine delivery, antibacterial agent, and wound healing. However, the current research on chitosan-based nanoparticles is not extensive enough. Elaborate research on the biological properties and preparation of chitosan and its derived nanoparticles should be a pressing need. In particular, the research on their toxicity to human beings should be comprehensively studied. Likewise, researchers should conduct in-depth studies on new usages for chitosan and also find out more about human-related effects through animal experiments. Chitosan and its derived nanoparticles will draw more and more attention and will have unlimited application prospects. However, we must consider environmental protection and green production in the development of chitosan-based high-tech products for applications in various fields for the benefit of humans.
